# Electrochemical Noise Measurement to Assess Corrosion of Steel Reinforcement in Concrete

**DOI:** 10.3390/ma14185392

**Published:** 2021-09-18

**Authors:** Douglas Mills, Paul Lambert, Shengming Yang

**Affiliations:** 1 Waterside Campus, University of Northampton, Northampton NN1 5PH, UK; douglas.mills@northampton.ac.uk (D.M.); shengmingyang35@gmail.com (S.Y.); 2 Centre for Infrastructure Maintenance, Materials & Engineering Research Institute, Sheffield Hallam University, Howard Street, Sheffield S1 1WB, UK

**Keywords:** concrete, corrosion, electrochemical noise, reinforcement, steel

## Abstract

The electrochemical noise method (ENM) has previously been employed to monitor the corrosion of steel reinforcement in concrete. The development of solid-state Ag/AgCl-based probes and dedicated monitoring technology (ProCoMeter) now offers a wider range of ENM configurations. The present study involves the laboratory investigation of three mortar samples containing steel bars and varying additions of chloride, with a view to future field application. ENM could be used to provide corrosion information on reinforcement without the need to provide direct electrical connections to the steel and without the risk or inducing or increasing corrosion. In addition to half-cell potentials, measurements were made using ENM in three different probe configurations over a total test period of 90 days. The samples were then broken open and the bars extracted and cleaned. A comparison was then made between the calculated metal thickness loss obtained from the Rn values and the actual metal thickness loss. The results showed that each configuration was able to order the results in the expected manner, with the simple single substrate (SSS) arrangement providing the best correlation with direct measurements. The study is ongoing with the intention of measurements being obtained in situ on existing reinforced concrete structures.

## 1. Introduction

Electrochemical techniques to assess the corrosion of steel in concrete have been used with varying success for many years. A 2009 review of critical chloride levels by Angst et al. [1] compared the results from a large number of researchers using a range of methods to determine the onset of corrosion. The review identified the three most popular methods employed were half-cell potential (45%), linear polarisation resistance (LPR) (41%), and visual assessment (24%), with the latter being destructive and thus of limited application in the field. The following is a summary of selected papers directly relevant to the current investigation and highlights the lack of recent studies into the use of electrochemical noise as a method of determining corrosion in reinforced concrete.

Electrochemical impedance spectroscopy (EIS) is a popular electrochemical technique among researchers and, in theory, can be used to assess reinforcement in concrete, although Videm [2] showed that there were difficulties in its use. Moreover, it has the disadvantage of being time-consuming because obtaining corrosion-related data requires low frequencies, which take a long time to measure. Back in 1988, early work on the use of the electrochemical noise method (ENM) was carried out by Hardon et al. [3], which also looked at potential mapping. It was pointed out that the potential map only provides an indication of the apparent corrosion possibility. It cannot directly generate local corrosion rates, and it cannot be used to provide such data without the development of additional mapping and computational models of concrete resistivity. Laboratory results obtained using ENM and LPR showed that the passive and corroding states could be distinguished, with the techniques correlating well. However, it was concluded at that time that electrochemical noise techniques would probably not be suitable for site investigation of structural corrosion because these effects can be masked by scatter unless precautions are taken to make sure that the measurements are made under stable environmental conditions. Although the Hardon paper was inconclusive, using noise in a laboratory situation proved more promising in work conducted fifteen years later by Mills et al. [4,5]. Again, the results obtained using noise measurements correlated well with the measurements obtained using linear polarisation resistance. However, ENM had the advantage that it could distinguish whether pitting or general corrosion was occurring by analysing the data set. In addition, as it is a passive system utilising no externally generated signal or polarising voltage, it does not risk influencing the corrosion behaviour of the steel. Overall, this work demonstrated that ENM has the capability to be an effective method to monitor the rate of corrosion of steel in concrete and should be suitable for field applications.

In the same year as the second Mills paper, Legat [6] compared ENM with measurements obtained from electrical resistance (ER) probes to study the time evolution of corrosion processes of steel in concrete, in order to evaluate the influence of concrete parameters on the corrosion process. These two techniques were used in the continuous wet and dry cycle of concrete specimens. The test results were compared with the corrosion evaluation results of the corroded steel. The results showed that both methods can effectively measure the corrosion of steel bars in concrete and have compatibility. Mariaca [7] analysed the ability of ENM to obtain quantitative information of corrosion rate of reinforcement in concrete. He concluded that electrochemical noise measurement seems to be an effective tool for estimating the corrosion rate of steel bars in concrete.

Recently published work by Garcia-Contreras et al. [8] and Permeh et al. [9] has used ENM techniques to monitor pitting corrosion in post-tensioned bridges and study the effect of fly ash additions on reinforcement subjected to cathodic protection, respectively. A review of electrochemical noise carried out in 2020 by Da-Hai Xia et al. [10] identified the numerous experimental arrangements and models that have been employed in an attempt to achieve near-quantitative evaluation of corrosion rates for a range of environments, including steel in concrete.

The above publications demonstrate that previous investigations that have evaluated the use of the ENM to monitor the corrosion of steel reinforcement in concrete have met some success, without ever getting to the point that ENM could be recommended as the method of choice for measurements in the field. Recently, the ENM technique has been improved further with the development of solid-state Ag/AgCl-based probes and the development of monitoring technology (ProCoMeter) designed for electrochemical noise measurements in the field, principally for assessing the effectiveness of organic coatings on metallic substrates, and offers two ENM configurations specifically designed to work effectively in a field situation and not previously available for use with steel in concrete. Relatively simple data treatment processes potentially enable more accurate results to be obtained than has been possible hitherto. Hence, it was considered appropriate to investigate the technique further using this recently developed equipment with ‘in field’ configurations and specially developed probes to assess its suitability for field application in the assessment of reinforced concrete structures.

The present study involves the laboratory investigation of three mortar samples containing steel bars and various chloride additions. The chloride reproduces the risk from mix contamination, admixtures, marine environments, or de-icing salts on the corrosion of reinforcement in concrete [11].

## 2. Materials and Methods

### 2.1. Sample Preparation

#### 2.1.1. Moulds 

These were polymer food containers with a sealed lid. The cover helped ensure adequate curing and maintained a moist environment. The capacity of the containers had a nominal volume of 6 L, ensuring the resultant specimens could be easily handled.

#### 2.1.2. Mortar

The mortar used for the samples as a simple model of concrete was a mix of sand (80%) and cement (20%) (approximate dimensions of box: 300 × 200 × 100 mm). A lean mix with a high water–cement ratio (e.g., 0.7) was employed to produce a relatively weak and permeable concrete in order to help accelerate the development of corrosion and assist with the breaking open of the specimens at the end of the experiments. One sample had no further additions (Sample A). To promote incipient anode behaviour in the second sample (Sample B), it was initially cast halfway with 4 wt.% Cl with a simple polystyrene form. The following day, the form was removed and the remaining was half cast with chloride free mortar directly against the 4 wt.% Cl half, reproducing a patch repair of chloride contaminated concrete with fresh material. To achieve relatively rapid corrosion initiation, the third sample (Sample C) was fully cast with 4 wt.% Cl added to the mix.

#### 2.1.3. Embedded Steel 

**Figure 1:**
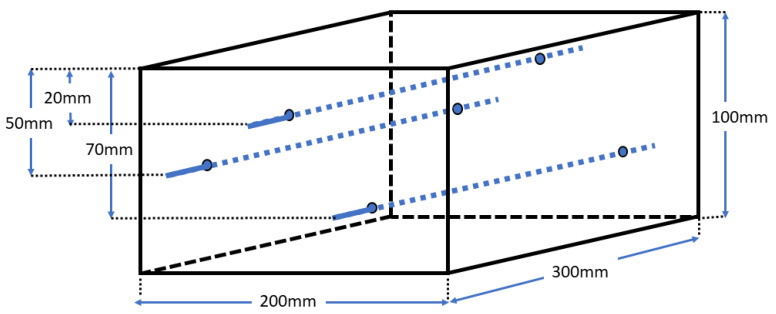
Schematic of mortar sample showing position of bars (not to scale).

To ensure that the mortar cover was not damaged by the expansive corrosion products, the diameter of steel needed to be relatively small, at 5.4 mm. The steel was a drawn wire with a 0.88% carbon content and an approximate UTS of 1800 MPa, and originally the straight central king wire from a seven-wire strand. The bars were formed by cropping king wires to be longer than the container used as the mould and passed through small holes in the container wall to hold them at the required cover depth. Each concrete sample has three bars at different cover depths, as shown in Figure 1.

#### 2.1.4. Samples 

Each sample had three steel bars running through it at different depths (Figure 1). As described in Section 2.1.2, the three samples contained different chloride additions. After initial curing, they were stored in a moist atmosphere in the sealed polymer containers in which they had been cast.

### 2.2. Test Methodology

#### 2.2.1. Half-Cell Potential Mapping 

**Figure 2:**
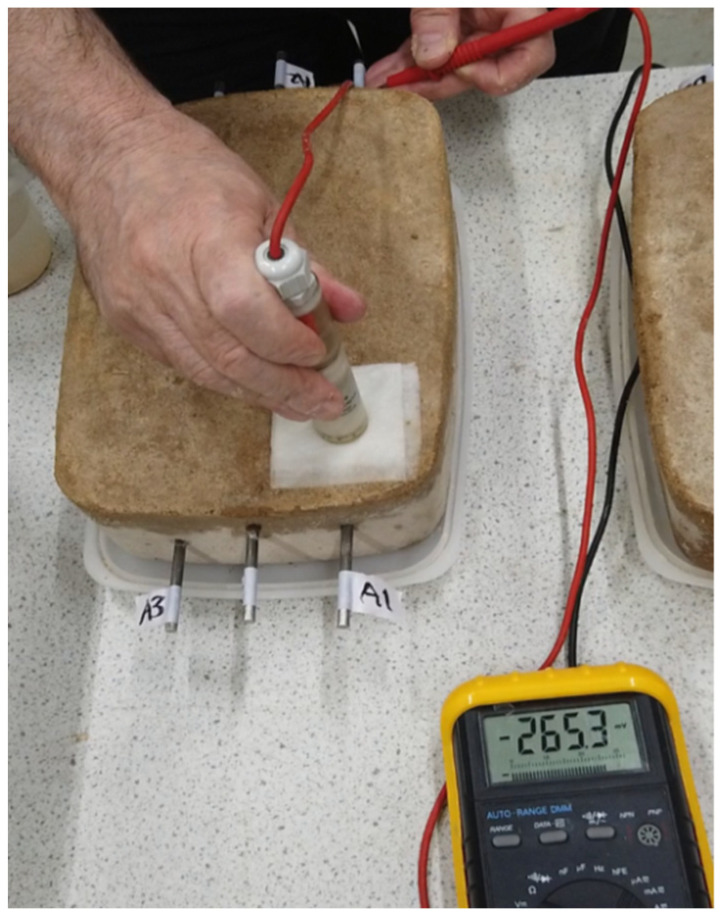
Half-cell potential mapping (Sample A).

Potential mapping is a common method used in the field [12,13,14]. The assumption made is that more negative values of potential (voltage) indicate a greater risk of corrosion, while less negative values of potential indicate a lower risk of corrosion. Before starting the measurements, the surface of concrete was wetted with a damp pad, as shown below in Figure 2. The reference electrode and bar were then connected to the negative and positive inputs of a digital voltmeter (DVM), respectively, and the potential was measured in millivolts DC. Three positions were measured above each bar. As each sample has three bars at different depths, nine values were collected from each sample (Table 1).

#### 2.2.2. Electrochemical Noise Method (ENM)

**Figure 3:**
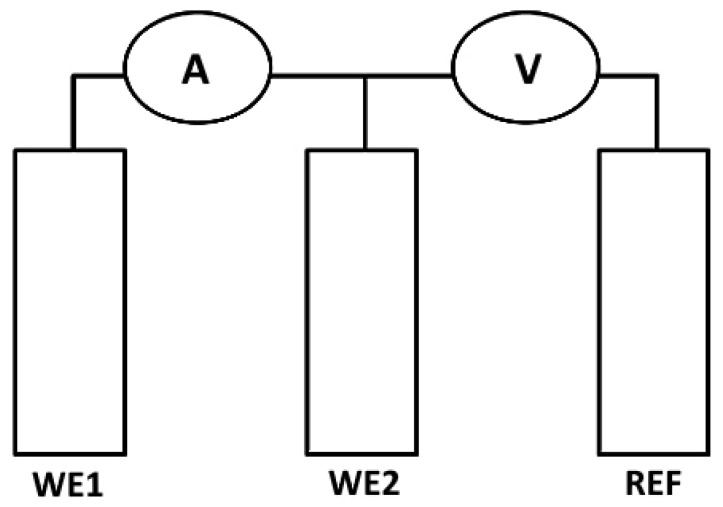
Schematic arrangement for ENM measurements.

The ENM system employed in this work always uses three electrode connections. The method involves the measurements of the small fluctuations in current signals that are created between two nominally identical electrodes, and the voltage signals that are generated between the reference electrode and the working electrodes. This is shown in Figure 3 and the method as applied to corrosion in solutions is described in ASTM G199 [15]. In the most common (Bridge) arrangement, there are two corroding ‘working electrodes’ (WE1, WE2) and a reference electrode (REF). If measuring in concrete or mortar, WE1 and WE2 are two reinforcement bars and WE3 is the probe, which is on the outside of the concrete above the two bars.

A further configuration can be used in which two pseudo reference electrodes (probes) act as working electrodes (connected to WE1 and WE2) and the bar itself becomes the reference (connected to REF). This is referred to as the simple single substrate arrangement (SSS). It is also possible to use just three reference electrodes (referred to as no connection to substrate or NOCS). Although these two latter configurations have been successfully applied to coatings [16], this is the first time they have been used to measure reinforcement in mortar or concrete.

In the current work, a dedicated ENM Instrument (ProCoMeter utilizing solid state reference electrodes, both supplied by the DCVG Company, Wigan, UK) was used and 2048 data points were gathered at five measurements per second. Each measurement was repeated twice, the data were logged, and then downloaded to a computer for analysis. The main parameter calculated was as follows:

(1) $$R_{n}=\frac{\mathrm{Standard Deviation of Voltage Noise}}{\mathrm{Standard Deviation of Current Noise}}$$

Rn can be related to corrosion current by the Stern–Geary equation:

R_p_ (or R_n_) = B/I_corr_, where B = B_a_ × B_c_/2.3 (B_a_ + B_c_) (2)

where R_p_ is the slope in ohms or ohms · cm^2^, I_corr_ is the corrosion current in amperes or amperes/cm^2^, and B_a_ and B_c_ are the Tafel constants in volts per decade of current.

It has been shown that Rn can be considered as equivalent to Rp [17]. Data treatment was sometimes required to obtain valid and accurate values of Rn. This might involve detrending, where any drift of the voltage or current is removed mathematically, or brushing, in which a limited section of the data set is selected to avoid jumps (but still sufficient to calculate Rn accurately), or both.

An important point to note is that, throughout this work, the assumption has been made that, where corrosion of the steel has taken place, this is predominantly general corrosion, as compared with pitting corrosion. This is confirmed by the lack of spikes in the data sets generated and the general appearance of the corroded bars at the end, which, although not completely uniform, shows the overall features of general corrosion. ENM has the ability to distinguish between pitting and general corrosion, as shown by the work reported in [4]. Ideally, the extent to which the data set is Gaussian could be checked by calculating skew and kurtosis values; however, the available software for use with the instrument did not offer this facility.

#### 2.2.3. Specific Arrangements Used for ENM 

**Figure 4:**
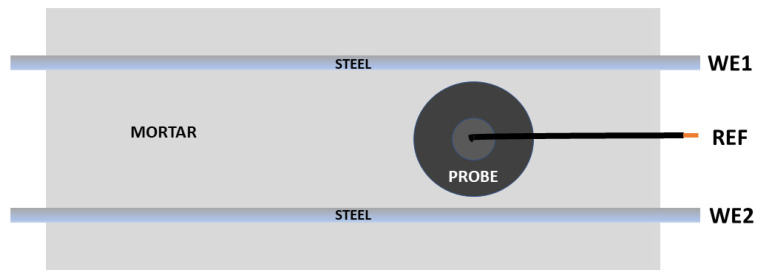
Schematic of bridge arrangement for ENM measurement.

**Figure 5:**
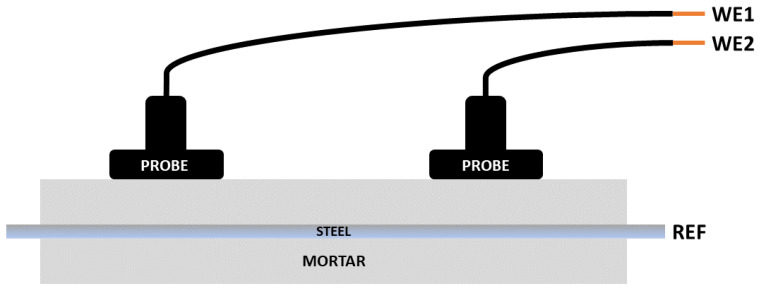
Schematic of simple single substrate (SSS) arrangement for ENM measurement.

**Figure 6:**
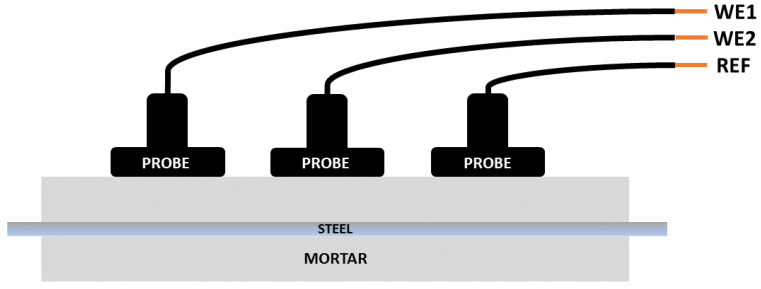
Schematic of no connection to substrate (NOCS) arrangement for ENM measurement.

As discussed earlier, the traditional arrangement for making ENM measurements (bridge mode) requires two nominally identical electrodes (WE1 and WE2) being interrogated at the same time, hence an electrical connection has to be made to two separate bars as shown in Figure 4. In the SSS mode shown in Figure 5, connection is only made to one bar (which becomes the pseudo reference) and the information obtained comes from just that one bar. The WEs in this mode become the two probes. In the NOCS mode shown in Figure 6, three probes (two of them acting as WEs and the other acting as REF) are used situated above one bar and there is no need to connect to the bar at all, which is a distinct advantage for field applications. One practical point is that the probes in SSS and NOCS mode should be placed at least three times the distance apart of the cover depth to avoid short circuiting [4].

#### 2.2.4. Probe Holder 

**Figure 7:**
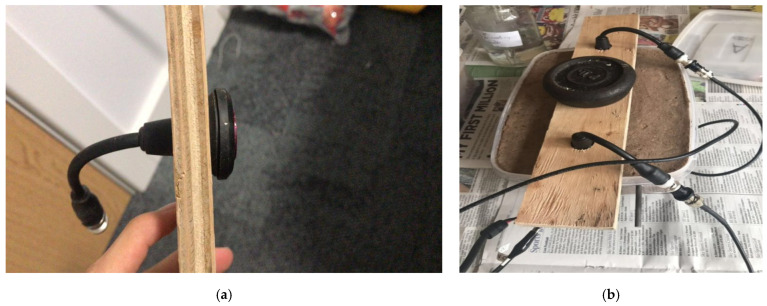
(**a**) Side view showing probe in place in probe holder. (**b**) Probe holder in use during SSS measurement.

This was designed to allow ENM measurements to be made using any of the three arrangements. It is shown in Figure 7a,b.

#### 2.2.5. Measurement Protocol 

During the curing period, half-cell potential measurements were taken, and after a period of two and half weeks, measurements were made using ENM on all three different arrangements sequentially (bridge, SSS, and NOCS). In the case of the bridge method, measurements were taken with the ref at both ends of the sample (near and far). The ENM method is totally unintrusive, so it was very unlikely that there would be any change in the corrosion processes during the time when the three different measurements were being made.

Before measurements were made, gentle wetting of the whole surface occurred using water. After the probe holder, probes, weight, and ProCoMeter were correctly assembled, the settings on the ProCoMeter were checked. It is necessary to keep the counter resistor within the recommended limits (normally 100 ohms to 10 K ohms). While measurements were being made, it was ensured that there was no interference to the results from the environment. These measurements continued periodically for a further eight weeks. The samples were kept in atmospheric conditions with the surface covered. The temperature varied from 5 °C at night to 25 °C at times during the day. The total test period was 90 days. Potential measurements were again obtained at the end of the test period prior to opening-up the specimens.

**Figure 8:**
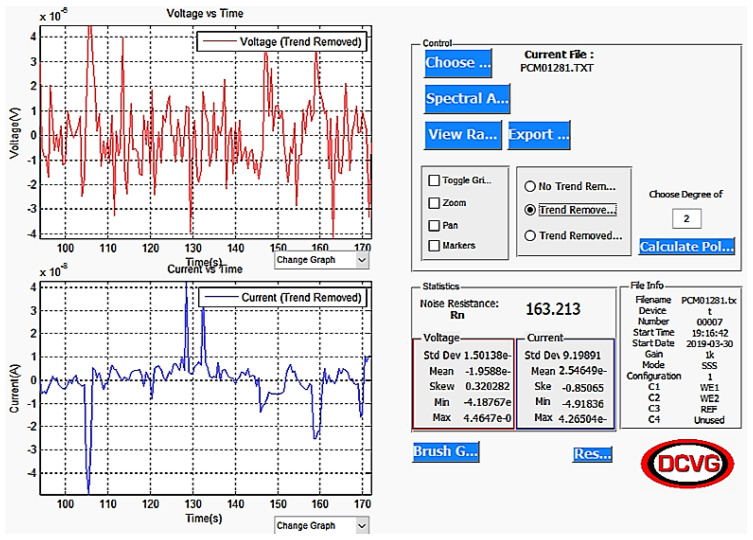
Example of ProCoMeter output using SSS arrangement.

When all ENM tests were completed, the data were downloaded from the ProCoMeter and analysed with software (brushing and detrending) to determine the values of Rn (Figure 8).

## 3. Results

### 3.1. Half-Cell Potential Measurements 

The results of the half-cell potential mapping given in Table 1 show a trend that is generally consistent with the age and exposure conditions of the bars, with those at the greatest risk of corrosion showing the most negative values.

Because there is no direct correlation between half-cell potential and corrosion rate, further evaluation of the corrosion is limited to that of ranking risk and cannot be used on its own to assess the rate at which the bars undergo section loss. This can only be properly assessed by exposing or removing the bars and measuring section loss directly, which has limited practicality when assessing the reinforcement condition in situ. The results of section loss assessments for the bars in the three samples are discussed later.

### 3.2. Electrochemical Noise Measurements 

**Figure 9:**
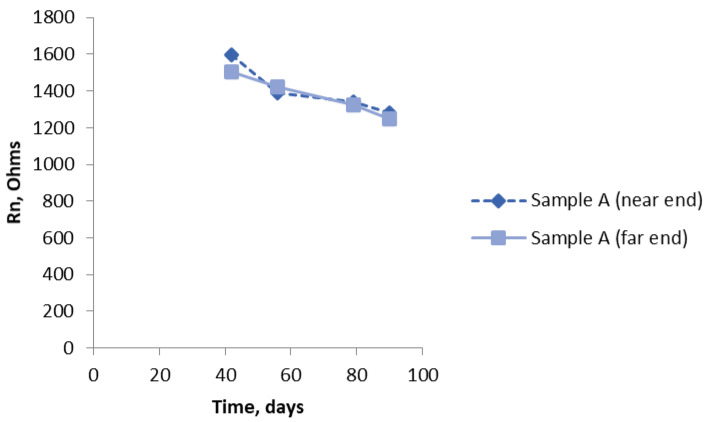
R_n_ results for Sample A (no chloride added) with bridge arrangement measured at both ends.

**Figure 10:**
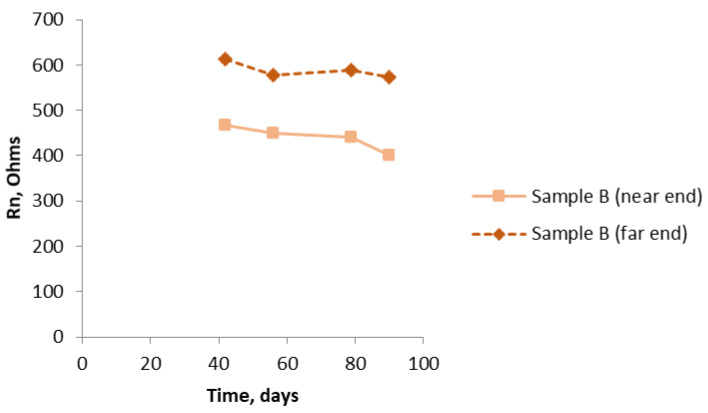
R_n_ results for Sample B (near end 4% Cl, far end 0% chloride) using the bridge arrangement.

**Figure 11:**
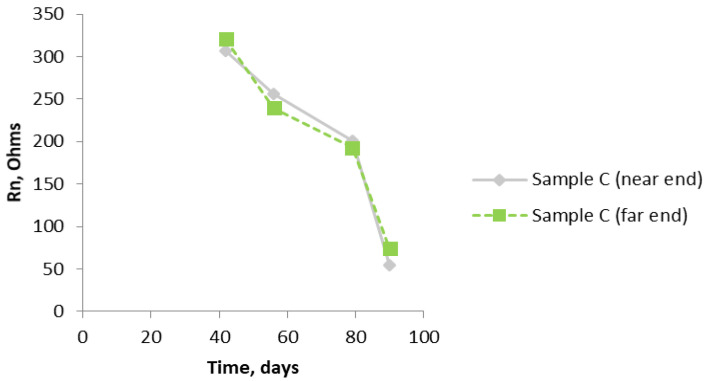
R_n_ results for Sample C (4% chloride levels throughout) using the bridge arrangement.

**Figure 12:**
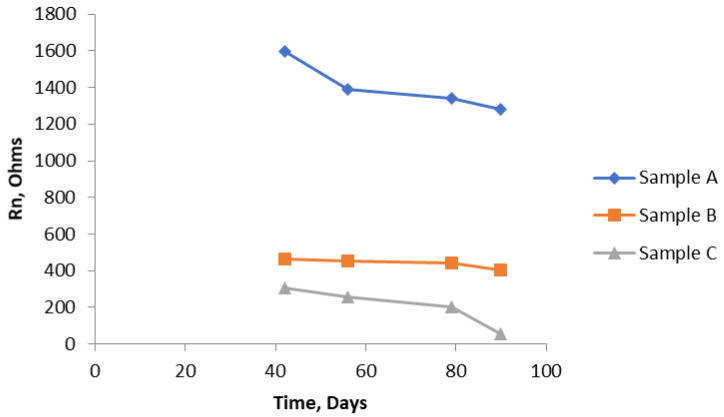
Comparison of R_n_ from all three samples (A, B, and C) with the bridge arrangement.

**Figure 13:**
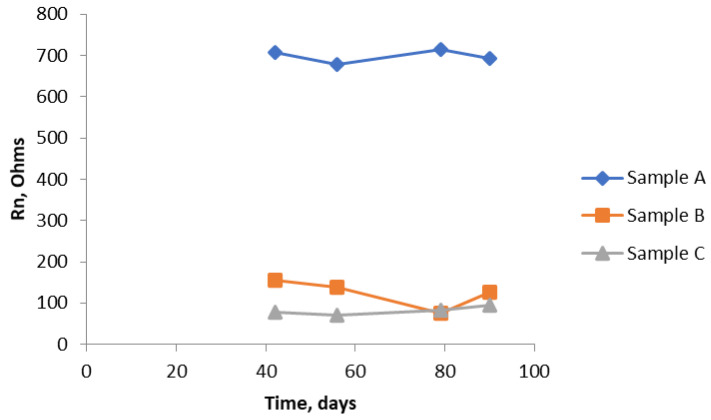
R_n_ results from all three samples (A, B, and C) using the SSS arrangement.

**Figure 14:**
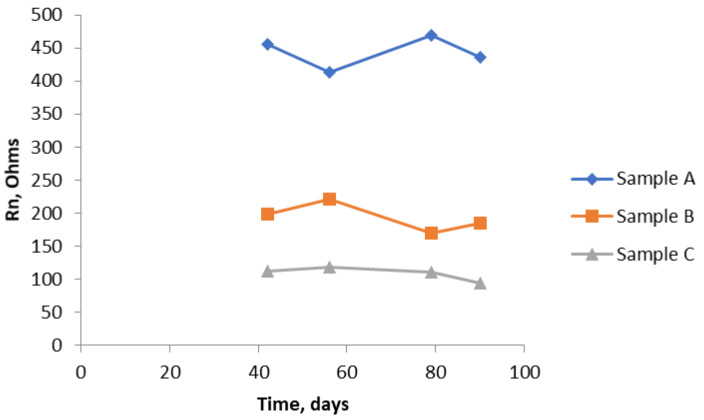
R_n_ results using NOCS arrangement for all three samples.

**Figure 15:**
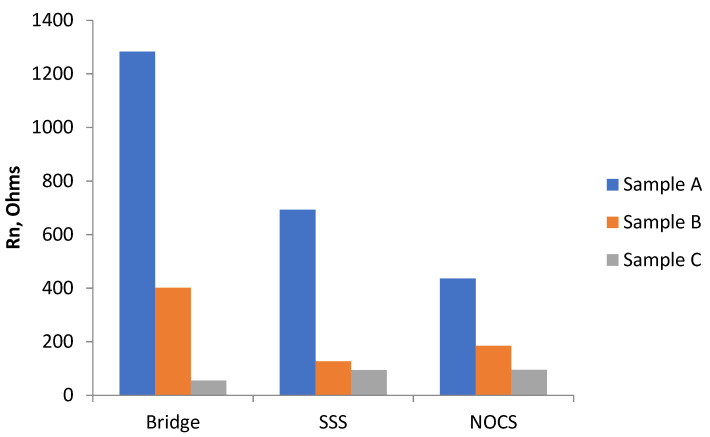
Comparison of R_n_ results obtained with the three ENM arrangements over the test period from 42 days to 90 days.

Graphical plots in Figure 9, Figure 10, Figure 11, Figure 12, Figure 13, Figure 14 and Figure 15 show the Rn values obtained. The data started being collected some way into the test period to allow some settling down. Figure 9 shows the trend downwards, but only slightly, of the values of Rn for Sample A and the results obtained when measuring at the two ends. There is excellent reproducibility and, as expected owing to the uniform nature of the composition of this sample, no significant difference between the two ends. Figure 10 shows a similar set of results for Sample B. Here, there is a distinct difference in the Rn results depending on which end of the sample was being interrogated (i.e., where the ref was located), with the end with chloride (near end) having significantly lower values of Rn. The overall values are less than they were for Sample A (about 1/3 to 1/4). Again, there is a moderate downward trend that is more noticeable in the end with chloride (note: the higher the Rn, the lower the corrosion rate).

Figure 11 shows that there is a dramatic drop in the values of Rn over the last fifty days of the test, showing that, by the end of the test, there is quite a significant corrosion rate of the bars. Again, as expected because of the uniform composition of the mortar, there is no significant difference between the two ends. The graph in Figure 12 brings all the results together for the near end (in the case of Sample B, this is the half of the sample with the chloride addition). Figure 13 shows similar results to Figure 12. They are unlikely to be exactly the same because, by using the SSS mode, just the uppermost bar is being interrogated in each of the three samples. The same trend is apparent in both graphs, although the differentiation between Sample B and Sample C is less clear cut. Owing to the limited size of the samples, it was not practicable to interrogate the two different ends of Sample B separately using the SSS mode, hence the Rn results for Sample B will be an amalgamation.

As shown in Figure 14, using the NOCS arrangement orders the three samples in the same way as the bridge and SSS arrangements. There is some degree of compression, in so far as Sample A has a somewhat lower value than one might expect from the results from the other two modes, although Sample A is still significantly higher.

To obtain an overall figure for the average corrosion rate, the average value of Rn over the measurement period was obtained. These results are shown in Table 2 and plotted as a bar chart in Figure 15. There is some scatter in these results as they are averages from the four different times. The degree of scatter is included in Table 2. These are likely to be underestimates, in so far as, in the first 42 days, no measurements were made, so the overall average might be higher, but they do enable some comparison to be made. They are used later to compare with the observed corrosion rates based on actual loss of metal.

The bar chart shown in Figure 15 shows the Rn values obtained from Sample A are much higher than Rn values of Sample B or Sample C, regardless of which of the three ENM measuring arrangements was used.

### 3.3. Examination of Bars at End of Test 

**Figure 16:**
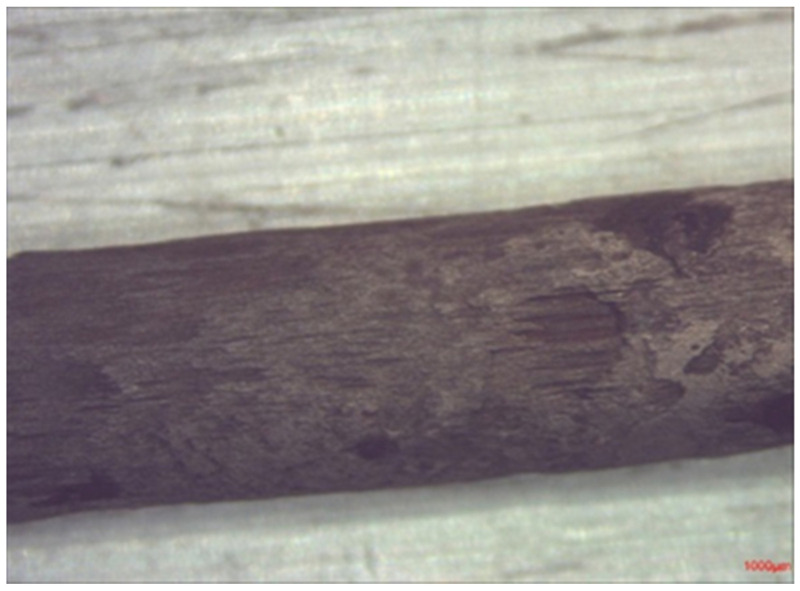
Close up of specimen C, 50 mm cover.

After three months, the samples were broken open and the bars were extracted. They were then immersed in 50% glacial acetic acid at room temperature for ten minutes, and then washed and dried. After cleaning, macro photos were taken of the bars and close ups of the corroded areas. The diameter of each bar was measured with a micrometer at different positions, with the greatest losses found on Sample C bars (Figure 16).

Table 3 gives the predicted metal thickness loss for the three bars using the average Rn values shown in Table 1 for the three ENM arrangements, (SSS, bridge, and NOCS). These values were obtained by applying Faraday’s Law: m = Mit/nF, conversion to section lost (d) using m = d·2πr·lρ, and the Stern–Geary equation I_corr_ = B/Rn using values of Ba and Bc of 120 mV/decade, typical for steel bars corroding in concrete, where m is mass loss, M is atomic mass, I is corrosion current, *t* is time in seconds, *n* is the number of electrons involved, F is the Faraday constant, r is the bar radius, and ρ is density.

The section loss at corroded areas was measured using a micrometer. By making an estimate of the area affected for each bar, this enabled the average loss of metal to be calculated over the whole period of exposure (90 days). The results are shown in Table 4.

## 4. Discussion

**Figure 17:**
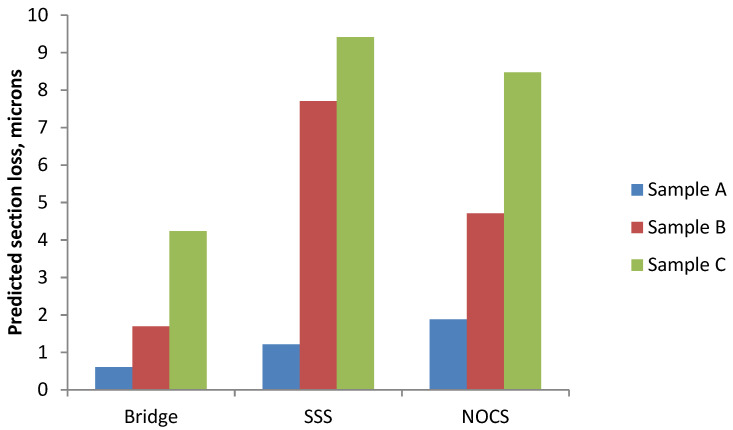
Predicted section loss for the three ENM methods based on R_n_ values.

The values of estimated steel section loss, based on the average Rn values given in Table 3 and shown graphically in Figure 17, range from 0.6 microns (Sample A using Bridge mode) to 9.4 microns (Sample C using SSS mode). For each mode/arrangement, the Rn results are in the order predicted from the level of chloride addition made to the mortar. The bridge method predicts lower metal loss than the other two (SSS and NOCS), with the latter two giving similar results.

Possible reasons for the differences in the section loss values obtained by the alternative arrangements are unclear, other than that there are different areas of the bars being interrogated by the bridge arrangement with two bars, compared with the SSS and NOCS arrangements each with one bar. With the NOCS mode, the section loss of Sample B is around twice that of Sample C. The bridge and SSS modes show a much larger difference between Sample B and Sample C, approaching eight times. It would be expected to see some difference between the 4% chloride sample and the half 4% chloride, half 0% chloride sample, but this large a difference is unexpected.

Comparing estimated section loss based on average Rn values and actual section loss, it was found that actual loss is somewhat higher in the case of Samples B and C, but somewhat lower in the case of Sample A.

The bridge method gives the lowest results for section loss. SSS and NOCS give similar results, with Sample C (uniform 4% Cl^−^) being indicated as having the highest loss and Sample A (0%Cl^−^) having the lowest.

## 5. Conclusions

The measurements carried out using steel bars in a simple cement mortar containing varying levels of chloride ions have demonstrated that dedicated proprietary ENM hardware and software originally developed for evaluating condition of coated steel can be effective in the monitoring of reinforcement corrosion. In addition to being easily portable and self-contained, the ProCoMeter allows configurations of working and reference electrodes that have not previously been employed with reinforced concrete.

It was possible to demonstrate that each of the three ENM modes available can order the results correctly, with the simple single substrate (SSS) arrangement giving the closest results to the direct section loss measurements for the steel bars undergoing significant rates of corrosion.

The no connection to substrate (NOCS) arrangement is of potentially greatest interest with respect to field evaluation, requiring no direct connection to the steel and thus avoiding the requirement to breakout and connect to the reinforcement. The results obtained are similar to the SSS arrangement and should thus be sufficiently accurate for field assessments.

The evaluation of ENM for assessing the corrosion condition in reinforced concrete applications utilising the ProCoMeter is presently ongoing. Several sites have been identified and are being evaluated for further in situ trials. The NOCS arrangement is considered particularly valuable for investigating structures incorporating multiple elements with little or no electrical continuity, such as precast panels and prestressed beams.

## Figures and Tables

**Table 1:** Half-cell potential results.

**Sample A—0% Cl**			
Cover	20 mm	50 mm	70 mm
Age	Half Cell Potential, mV vs. SSC 20 mm/50 mm/70 mm Cover (NT—not tested)		
3 days	−125/NT/−123	−130/NT/−129	−136/NT/−135
10 days	−082/NT/−079	−068/NT/−071	−085/NT/−080
90 days	−314/−317/−295	−266/−256/−269	−177/−173/−167
**Sample B—0% Cl (near) 4% Cl (far)**			
Cover	20 mm	50 mm	70 mm
Age	Half Cell Potential, mV vs. SSC 20 mm/50 mm/70 mm Cover (NT—not tested)		
3 days	−245/NT/−239	−342/NT/−330	−290/NT/−284
10 days	−172/NT/−166	−296/NT/−285	−322/NT/−318
90 days	−413/−452/−473	−443/−484/−489	−384/−414/−437
**Sample C—4% Cl**			
Cover	20 mm	50 mm	70 mm
Age	Half Cell Potential, mV vs. SSC 20 mm/50 mm/70 mm Cover (NT—not tested)		
3 days	−327/NT/−324	−358/NT/−356	−351/NT/−352
10 days	−374/NT/−353	−387/NT/−362	−390/NT/−378
90 days	−517/−532/−531	−515/−512/−496	−424/−435/−440

**Table 2:** Average Rn value over whole test period (ohms).

Measuring Arrangement	Sample A	Sample B	Sample C
SSS	700(+/−30)	110(+/−25)	90(+/−20)
BRIDGE	1400(+/−100)	500(+/−60)	200(+/−100)
NOCS	450(+/−25)	180(+/−20)	100(+/−10)

**Table 3:** Average calculated section loss (µm).

Sample	SSS	Bridge	NOCS	Average
A	1.2	0.6	1.9	1.2
B	7.7	1.7	4.7	4.7
C	9.4	4.2	8.5	7.4

**Table 4:** Measured localised section loss, area affected, and average section loss for bars in samples A, B, and C.

Sample	Localised Section Loss (µm)	Area Affected (%)	Average Section Loss (µm) over Whole Bar
A	11.8	1%	0.12
B	119	10%	11.9
C	98	20%	19.6

## Data Availability

The data underlying this article will be shared on reasonable request from the corresponding author.
